# Supplementary material for: Coordination chemogenetics for activation of GPCR-type glutamate receptors in brain tissue
Source: Nat Commun. 2022 Jun 16;13:3167. doi: 10.1038/s41467-022-30828-0 (PMC9203742; doi:10.1038/s41467-022-30828-0)
Supplement: Supplementary file 1 — Supplementary Information [file 41467_2022_30828_MOESM1_ESM.pdf]

# SUPPLEMENTARY INFORMATION

## Coordination chemogenetics for activation of GPCR-type glutamate receptors in brain tissue

Kento Ojima[1,2#], Wataru Kakegawa[3#], Tokiwa Yamasaki[3], Yuta Miura[1], Masayuki Itoh[3], Yukiko Michibata[2], Ryou Kubota[2], Tomohiro Doura[1], Eriko Miura[3], Hiroshi Nonaka[2], Seiya Mizuno[4], Satoru Takahashi[4], Michisuke Yuzaki*[3], Itaru Hamachi*[2], Shigeki Kiyonaka*[1,5]

[1]Department of Biomolecular Engineering, Graduate School of Engineering, Nagoya University, Nagoya 464-8603, Japan

[2]Department of Synthetic Chemistry and Biological Chemistry, Graduate School of Engineering, Kyoto University, Kyoto 615-8510, Japan

[3]Department of Neurophysiology, Keio University School of Medicine, Tokyo 160-8582, Japan

[4]Laboratory Animal Resource Center, Faculty of Medicine, University of Tsukuba, Tsukuba 305-8575, Japan.

[5]Institute of Nano-Life-Systems, Institutes of Innovation for Future Society, Nagoya University, Nagoya 464-8603, Japan.

*Correspondence: myuzaki@keio.jp
ihamachi@sbchem.kyoto-u.ac.jp
kiyonaka@chembio.nagoya-u.ac.jp

[#]These authors contributed equally to this work.

**Supplementary Table 1 | Distance (Å) between α-carbon atoms estimated by the crystal structure of mGlu1 open form (PDB 1EWT) and closed form (PDB 1EWK).**

| | H54-N264 | H55-N264 | E60-N264 | H111-N264 |
|---|---|---|---|---|
| Apo | 20.1 | 16.3 | 16.8 | 17.4 |
| Glutamate binding | 13.9 | 10.2 | 10.4 | 10.9 |

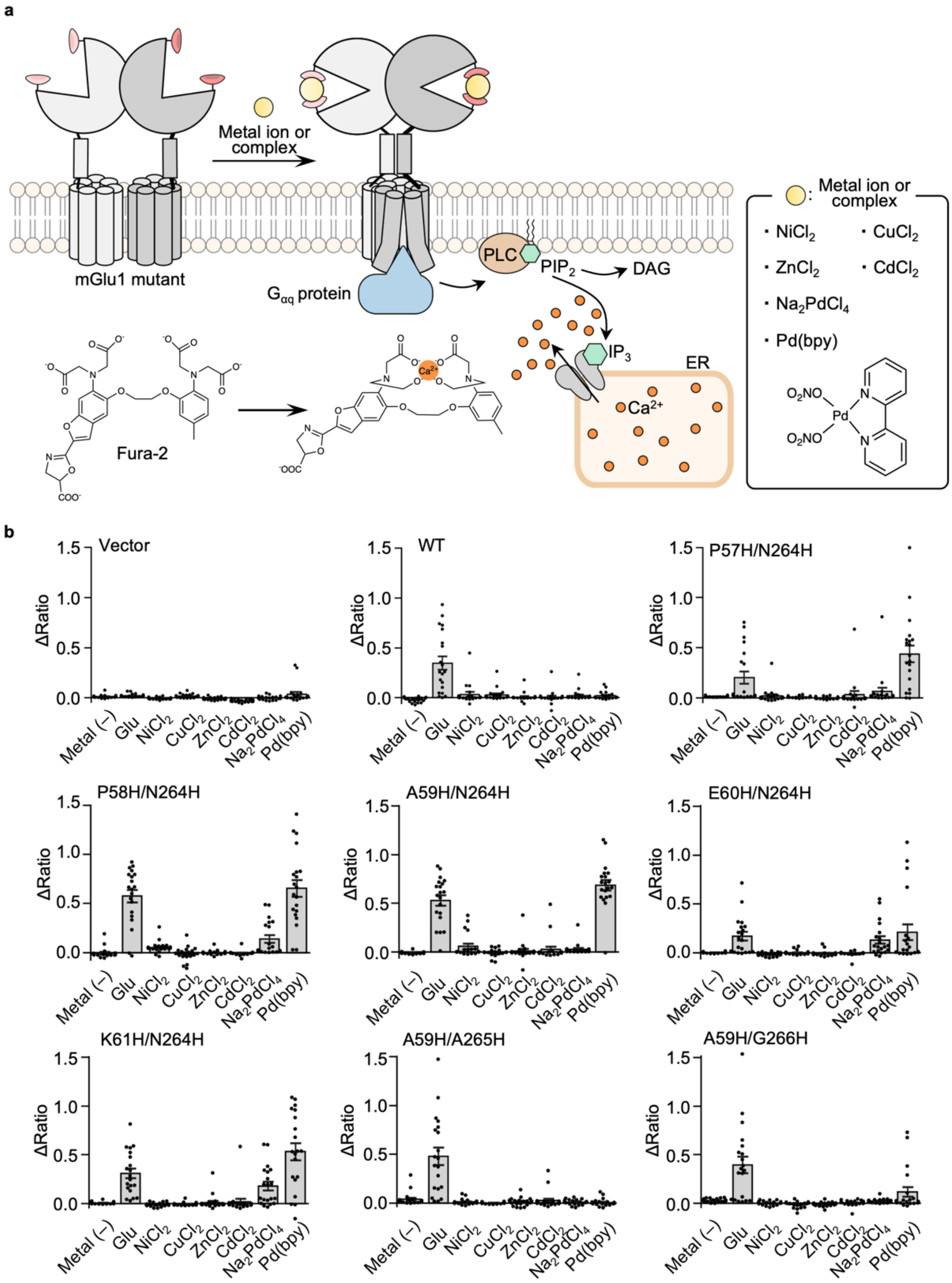


**Supplementary Figure 1 | Screening assay for mGlu1 double mutants with metal ions and a metal complex. (a)** Schematic illustrations of detection of $[Ca^{2+}]_i$ changes after activation of Gq-coupled mGlu1 using Fura-2. **(b)** Averaged Δratio induced by 10 µM of glutamate, metal ions, or metal complex. (n = 20). Data are presented as mean ± s.e.m.

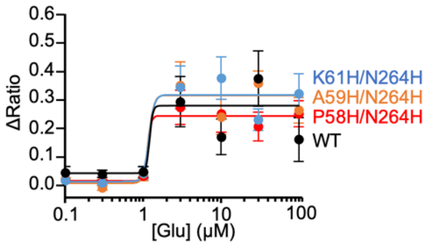


**Supplementary Figure 2 | Concentration-dependent curves of glutamate for hit mutants or WT mGlu1.** Concentration-dependent curves for glutamate in HEK293 cells expressing mGlu1 WT (black), P58H/N264H (red), A59H/N264H (orange) or K61H/N264H (blue). (n = 20). Data are presented as mean ± s.e.m.

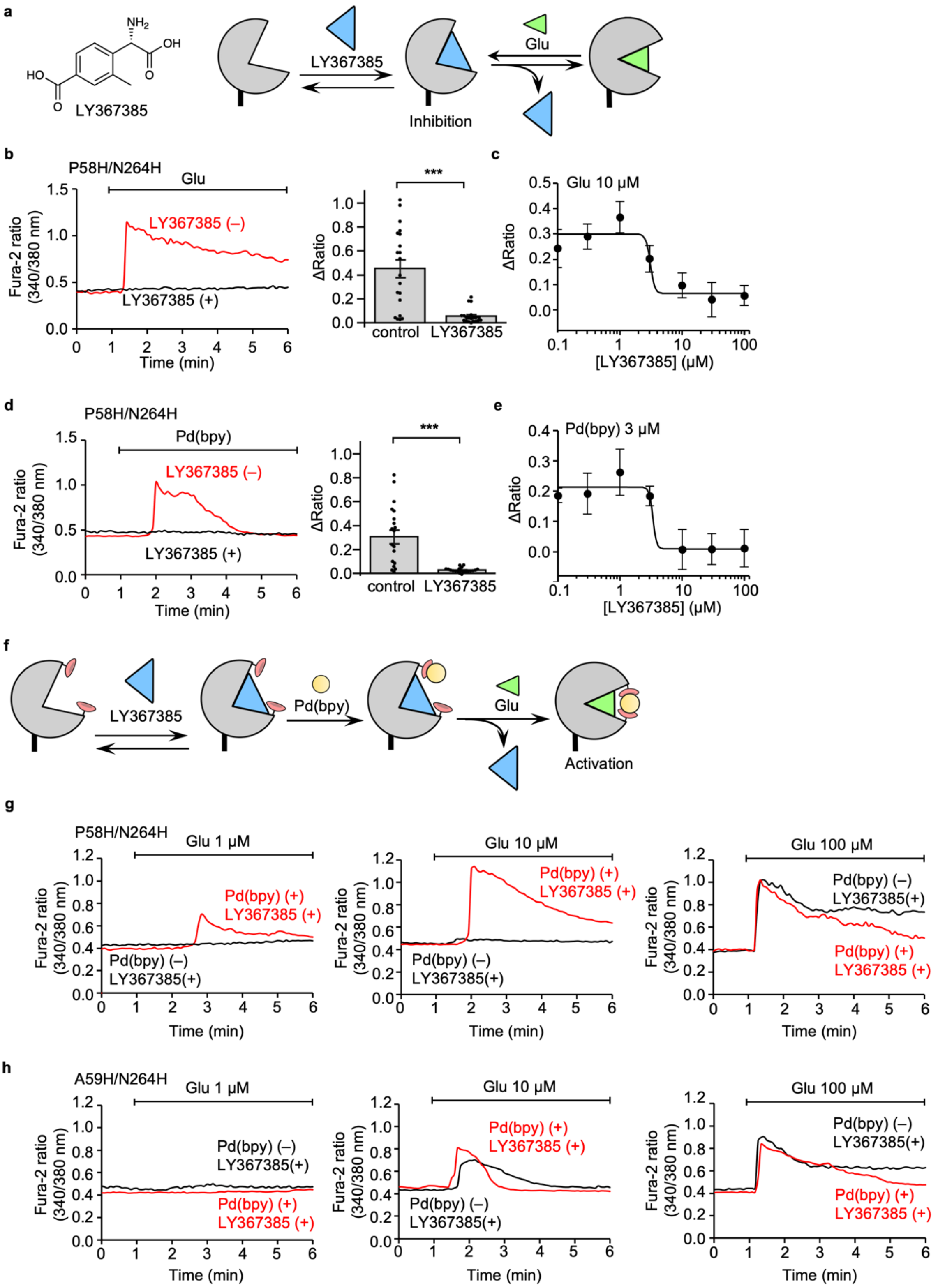


**Supplementary Figure 3 | Evaluation of positive allosteric effects of the Pd(bpy) for mGlu1 mutants by co-treatment of competitive antagonist, LY367385. (a)** Schematic illustrations of competitive inhibition of mGlu1 using LY367385. (**b, c**) Inhibition of the 10 μM glutamate-induced responses using LY367385 in HEK293 cells transfected with the plasmid of mGlu1 P58H/N264H mutant. In **b**, representative traces of $Ca^{2+}$ response with (black) or without (red) 10 μM LY367385 are shown (n = 20), and averaged Δratio is shown in the right. $P = 4.296 \times 10^{-5}$. (Two-tailed Welch's t-test, $***P < 0.001$). In **c**, a concentration-dependent curve of LY367385 for the inhibition of 10 μM glutamate-induced response is shown. (n = 20). (**d, e**) Inhibition of the 3 μM Pd(bpy)-induced responses using LY367385 in HEK293 cells transfected with the plasmid of mGlu1 P58H/N264H mutant. In **d**, representative traces of $Ca^{2+}$ response with (black) or without (red) 10 μM

LY367385 are shown (n = 20), and averaged Δratio is shown in the right. $P = 1.172 \times 10^{-4}$. (Two-tailed Welch's t-test, $***P < 0.001$). In **e**, a concentration-dependent curve of LY367385 for the inhibition of 3 μM Pd(bpy)-induced response is shown. (n = 20). (**f**) Schematic illustrations of the evaluation of positive allosteric effect of Pd(bpy) for glutamate-induced responses in the presence of LY367385. (**g, h**) Representative trace of glutamate-induced $Ca^{2+}$ response with (black) or without (red) 10 μM Pd(bpy) in the presence of 10 μM of LY367385 in HEK293 cells transfected with the plasmid of mGlu1 P58H/N264H (in **g**) or A59H/N264H (in **h**) mutant. Data are presented as mean ± s.e.m.

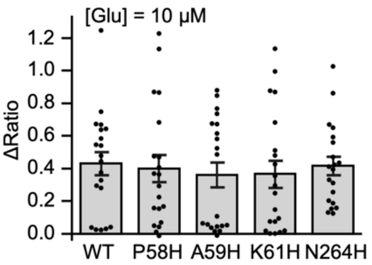


**Supplementary Figure 4 | Averaged Δratio induced by 10 μM of glutamate for mGlu1 WT and single mutants.** Glutamate-induced mGlu1 responses were evaluated in HEK293 cells expressed with WT mGlu1, mGlu1(P58H), mGlu1(A59H), mGlu1(K61H), or mGlu1(N264H) mutant. (n = 20). Data are presented as mean ± s.e.m.

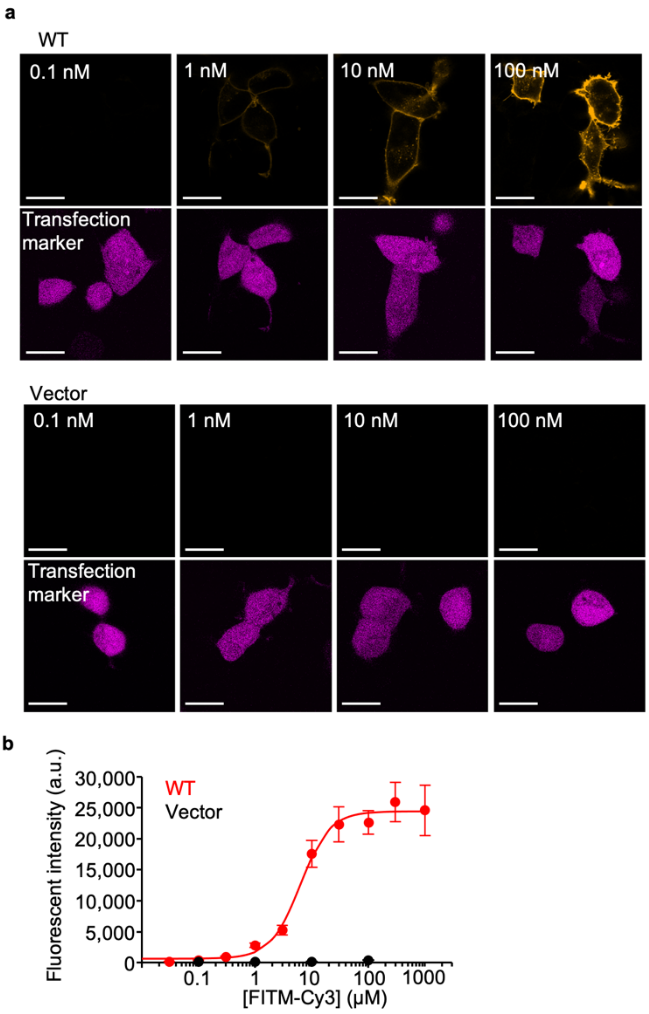


**Supplementary Figure 5 | Evaluation of the affinity of FITM-Cy3 to mGlu1 WT by confocal microscopy.** (**a**) Confocal live imaging of surface mGlu1 using various concentrations of FITM-Cy3 (upper) and transfection marker (lower) in HEK293 cells expressing WT mGlu1 or the control vector. iRFP670 was utilized as a transfection marker. Scale bars, 20 µm. (**b**) Concentration-dependent curves for fluorescent intensity of FITM-Cy3 in HEK293 cells expressing WT mGlu1 (red) and vector control (black) (n= 26–118). The $K_d$ value of FITM-Cy3 to WT mGlu1 on the cell-surface was determined to be 6.8 ± 2.7 nM (n = 3, biologically independent experiments). Data are presented as mean ± s.e.m.

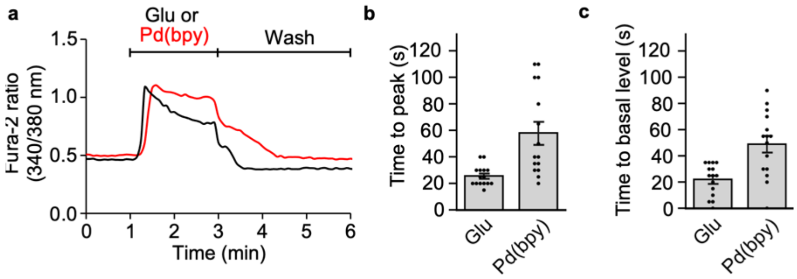


**Supplementary Figure 6 | The kinetics of Pd(bpy)-induced responses in the mGlu1(N264H) mutant.** (**a**) Representative traces of $Ca^{2+}$ response of 10 μM Glu (black) or 10 μM Pd(bpy) (red) in HEK293 cells expressing mGlu1(N264H) are shown. (**b**, **c**) Time to peak (in **b**) and time to basal level after washing by culture medium (in **c**) are shown. (n = 15). Data are presented as mean ± s.e.m.

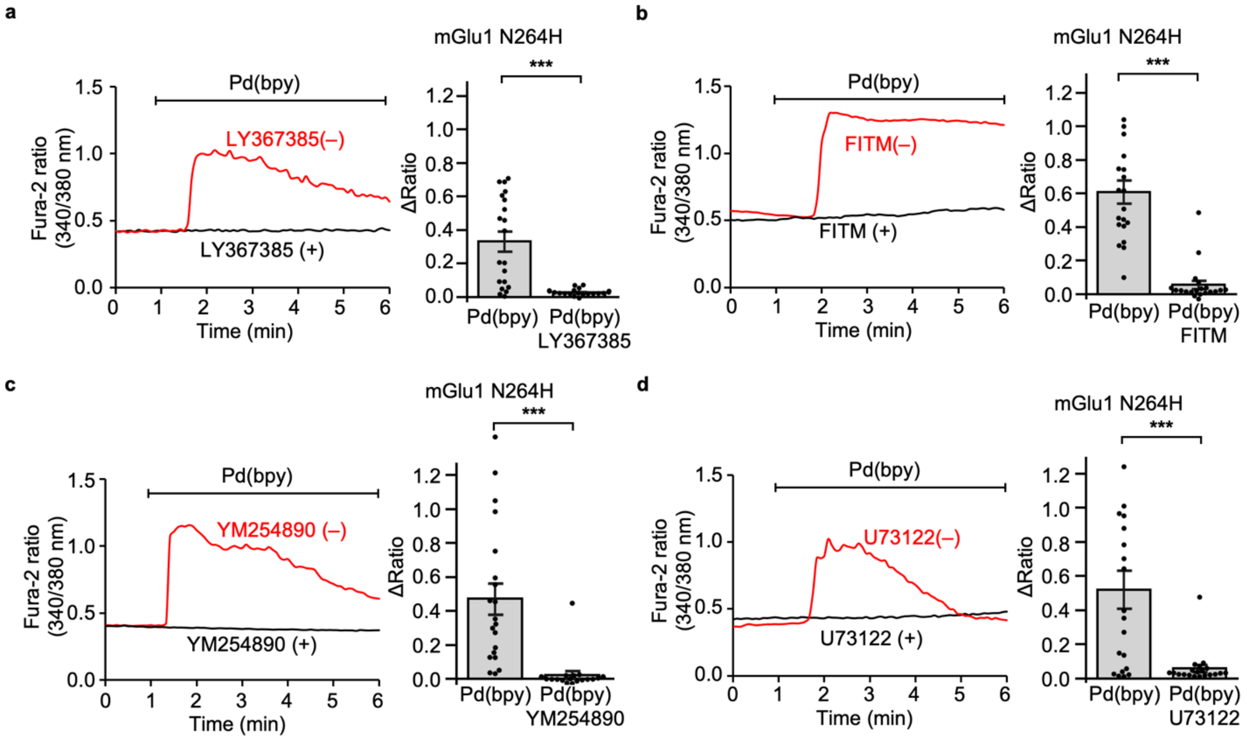


**Supplementary Figure 7 | Inhibition of Pd(bpy) induced-$Ca^{2+}$ responses using LY367385, FITM, or U73122.** Left: Representative trace of 10 µM Pd(bpy)-induced responses pre-treated with 10 µM LY367385 (in **a**), 1 µM FITM (in **b**), 10 µM YM254890 (in **c**) or 1 µM U73122 (in **d**) in HEK293 cells expressing mGlu1(N264H). Right: averaged Δratio. (n = 20). $P = 6.032 \times 10^{-5}$ in **a**, $9.185 \times 10^{-8}$ in **b**, $1.171 \times 10^{-4}$ in **c**, $5.997 \times 10^{-4}$ in **d**. (Two-tailed Welch's t-test, $^{***}P < 0.001$). Data are presented as mean ± s.e.m.

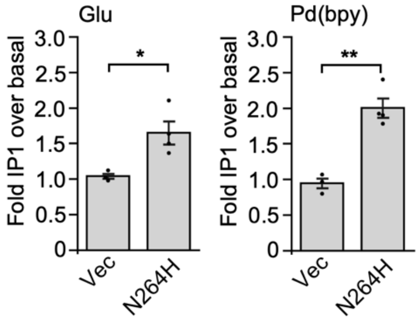


**Supplementary Figure 8 | Measurement of IP1 accumulation via Gq-pathway.** Left: 100 μM Glu-induced IP1 accumulations in HEK293 cells expressing mGlu1(N264H). Right: 30 μM Pd(bpy)-induced IP1 accumulations in HEK293 cells expressing mGlu1(N264H). (n = 3 or 4 for vector or mGlu1(N264H), respectively). $P = 0.03501, 0.00112$ for Glu, Pd(bpy), respectively. (Two-tailed Welch's t-test, $^{*}P < 0.05$, $^{**}P < 0.01$). Data are presented as mean ± s.e.m.

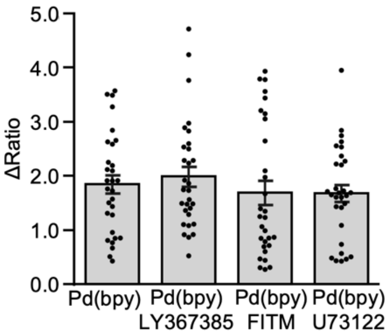


**Supplementary Figure 9 | The abnormal $Ca^{2+}$ response of Pd(bpy) was inhibited by neither mGlu1 inhibitors nor a PLC inhibitor in the cultured cortical neurons.** Averaged Δratio of 30 μM Pd(bpy)-induced responses in cultured cortical neuron co-treated with 30 μM LY367385, 1 μM FITM, or 10 μM U73122. (n = 30). Data are presented as mean ± s.e.m.

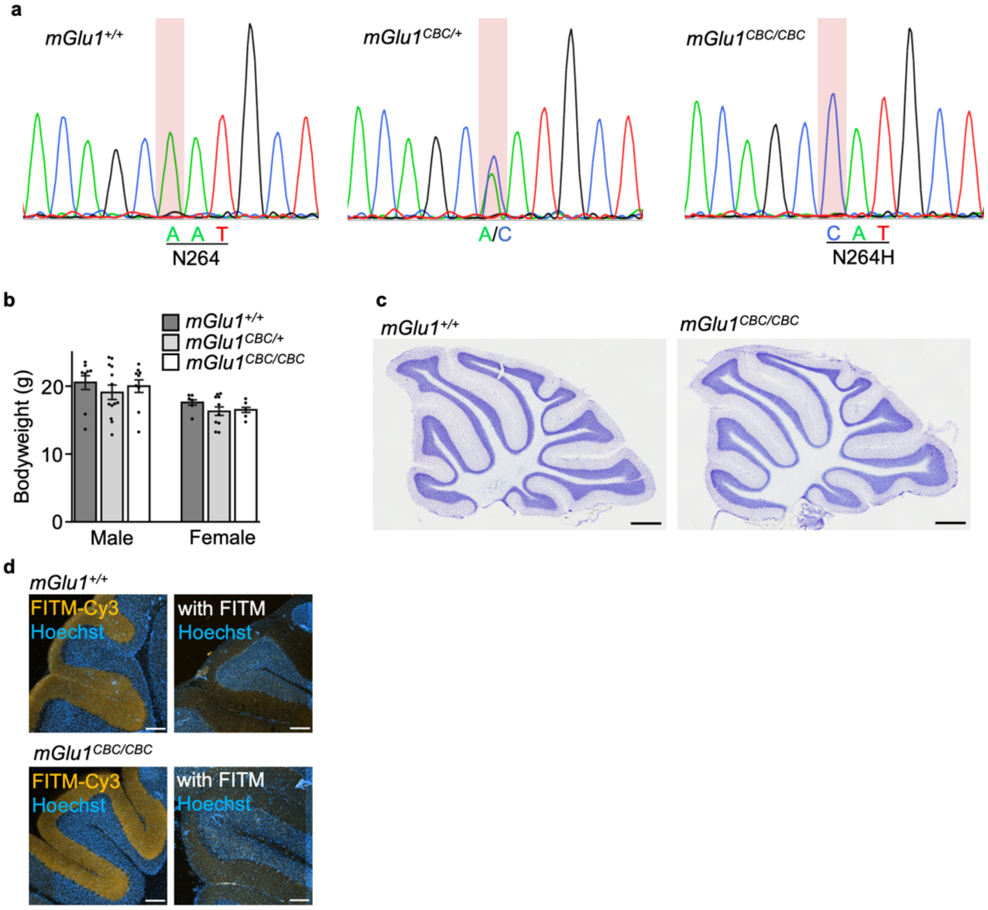


**Supplementary Figure 10 | Evaluation of functionality of the mGlu1 knock-in mouse.** (**a**) Sequence analyses of exon 3 of *mGlu1* using *mGlu1*$^{+/+}$, *mGlu1*$^{CBC/+}$, or *mGlu1*$^{CBC/CBC}$ genome. (**b**) Bodyweight of the *mGlu1*$^{+/+}$, *mGlu1*$^{CBC/+}$ and *mGlu1*$^{CBC/CBC}$ mice (6 weeks old). (n = 8, 13, or 9 for *mGlu1*$^{+/+}$, *mGlu1*$^{CBC/+}$, or *mGlu1*$^{CBC/CBC}$ -male, respectively. n = 6, 10, or 5 for *mGlu1*$^{+/+}$, *mGlu1*$^{CBC/+}$, or *mGlu1*$^{CBC/CBC}$ -female, respectively.) (**c**) Cresyl violet-stained sagittal sections of adult *mGlu1*$^{+/+}$ and *mGlu1*$^{CBC/CBC}$ mouse cerebella. Scale bar, 500 µm. Data are presented as mean ± s.e.m. (**d**) Staining of acute cerebellar slices of *mGlu1*$^{+/+}$ and *mGlu1*$^{CBC/CBC}$ using FITM-Cy3 and Hoechst33342. Scale bars, 200 µm. (n = 5)

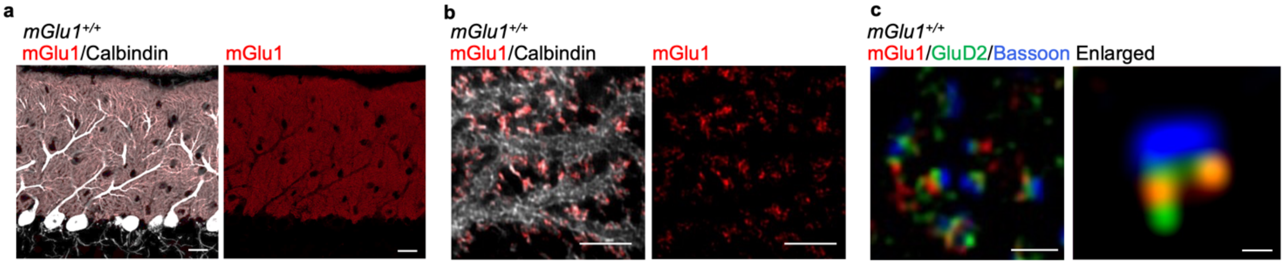


**Supplementary Figure 11 | Immunohistochemistry using anti-mGlu1 antibody in *mGlu1*$^{+/+}$ cerebellar slice.** (**a**, **b**) Conventional confocal (**a**) and super-resolution (**b**) microscopy images of immune-positive signals for mGlu1 (red) and calbindin (white) in the molecular layer of a *mGlu1*$^{+/+}$ cerebellar slice. Scale bars, 20 µm (in **a**) and 2 µm (in **b**). (**c**) Super-resolution microscopic images of immune-positive signals for mGlu1 (red), GluD2 (green) and Bassoon (blue). Scale bar, 1 µm. An enlarged view is shown in a right panel. Scale bar, 200 nm.

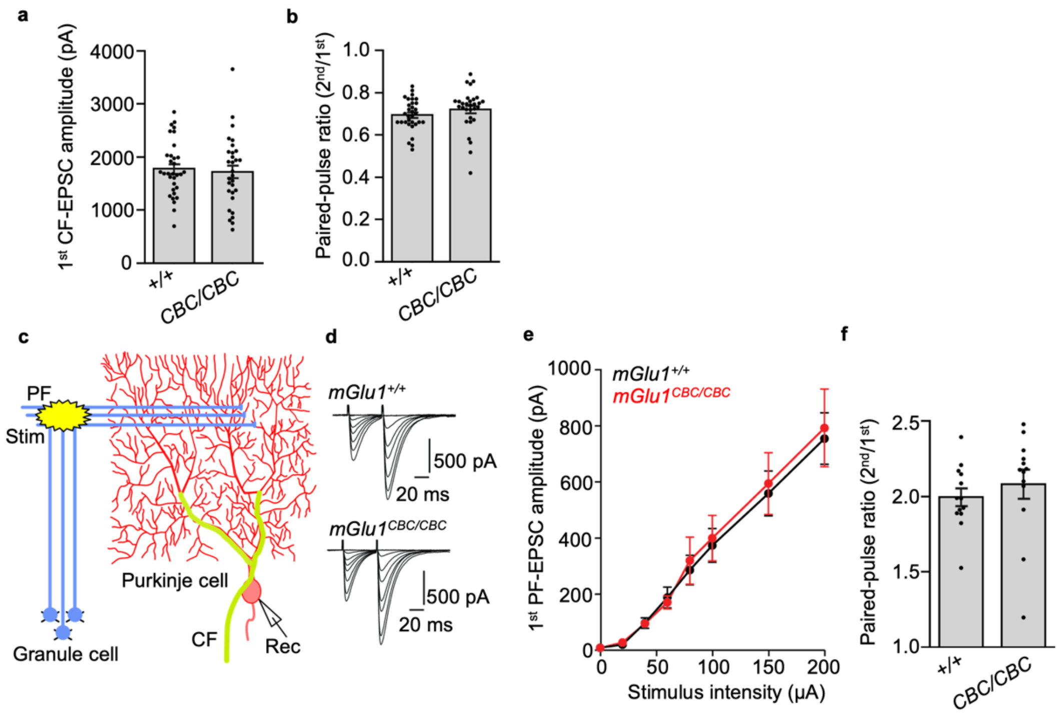


**Supplementary Figure 12 | Basic synaptic transmission is normal at CF and PF−Purkinje cells in *mGlu1$^{CBC/CBC}$* mice.** (**a**, **b**) Histograms showing CF-EPSC amplitude (**a**) and a paired-pulse ratio of CF-EPSC amplitudes (The amplitude of 2$^{nd}$ EPSC is normalized by that of 1$^{st}$ EPSC) in *mGlu1$^{+/+}$* (+/+, n = 32 cells from 5 mice) and *mGlu1$^{CBC/CBC}$* (CBC/CBC, n = 30 cells from 5 mice). *P* = 1.000 in **a** and 0.934 in **b** by two-sided Mann-Whitney U test. (**c, d**) An orientation of stimulus and recording electrodes (in **c**) to evoke PF-EPSCs (in **d**). In these experiments, EPSCs were evoked by the paired-pulse stimulation with various stimulus intensities (0, 20, 40, 60, 80, 100, 150, 200 μA; 50-ms inter-stimulus interval). (**e, f**) Input−Output relationship (in **e**) and paired-pulse ratio (in **f**) of the PF-EPSCs from *mGlu1$^{+/+}$* (n = 13 cells from 3 mice) or *mGlu1$^{CBC/CBC}$* (n = 13 cells from 3 mice, *P* = 0.822 by two-way repeated ANOVA in (**e**) and *P* = 0.101 by two-sided Mann-Whitney U test in (**f**)). Data are represented as mean ± s.e.m.

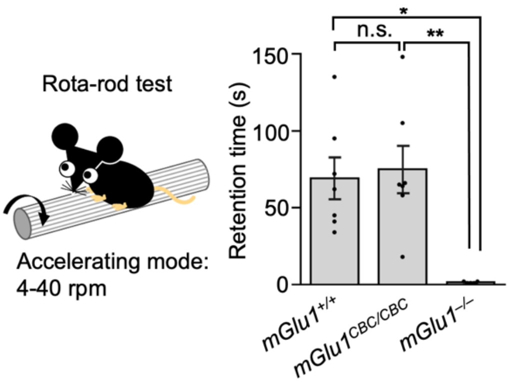


**Supplementary Figure 13 | Motor coordination is normal in *mGlu1*$^{CBC/CBC}$ mice.** To evaluate motor coordination of the mouse, accelerating rota-rod test (4−40 rpm for 5 min) was performed. Histogram shows averaged retention time on rota-rod in each mouse groups. High performance was observed in *mGlu1*$^{+/+}$ and *mGlu1*$^{CBC/CBC}$ mice but not *mGlu1*$^{-/-}$ mice. (n = 7 for *mGlu1*$^{+/+}$ and *mGlu1*$^{CBC/CBC}$. n = 5 for *mGlu1*$^{-/-}$). $P$ = 0.946, 0.0236, 0.0098 for *mGlu1*$^{+/+}$ vs *mGlu1*$^{CBC/CBC}$, *mGlu1*$^{+/+}$ vs *mGlu1*$^{-/-}$, *mGlu1*$^{CBC/CBC}$ vs *mGlu1*$^{-/-}$, respectively. (Kruskal-Wallis test followed by the Scheffe *post hoc* test, $^{**}P < 0.01$, $^{*}P < 0.05$, n.s. not significant). Data are presented as mean ± s.e.m.

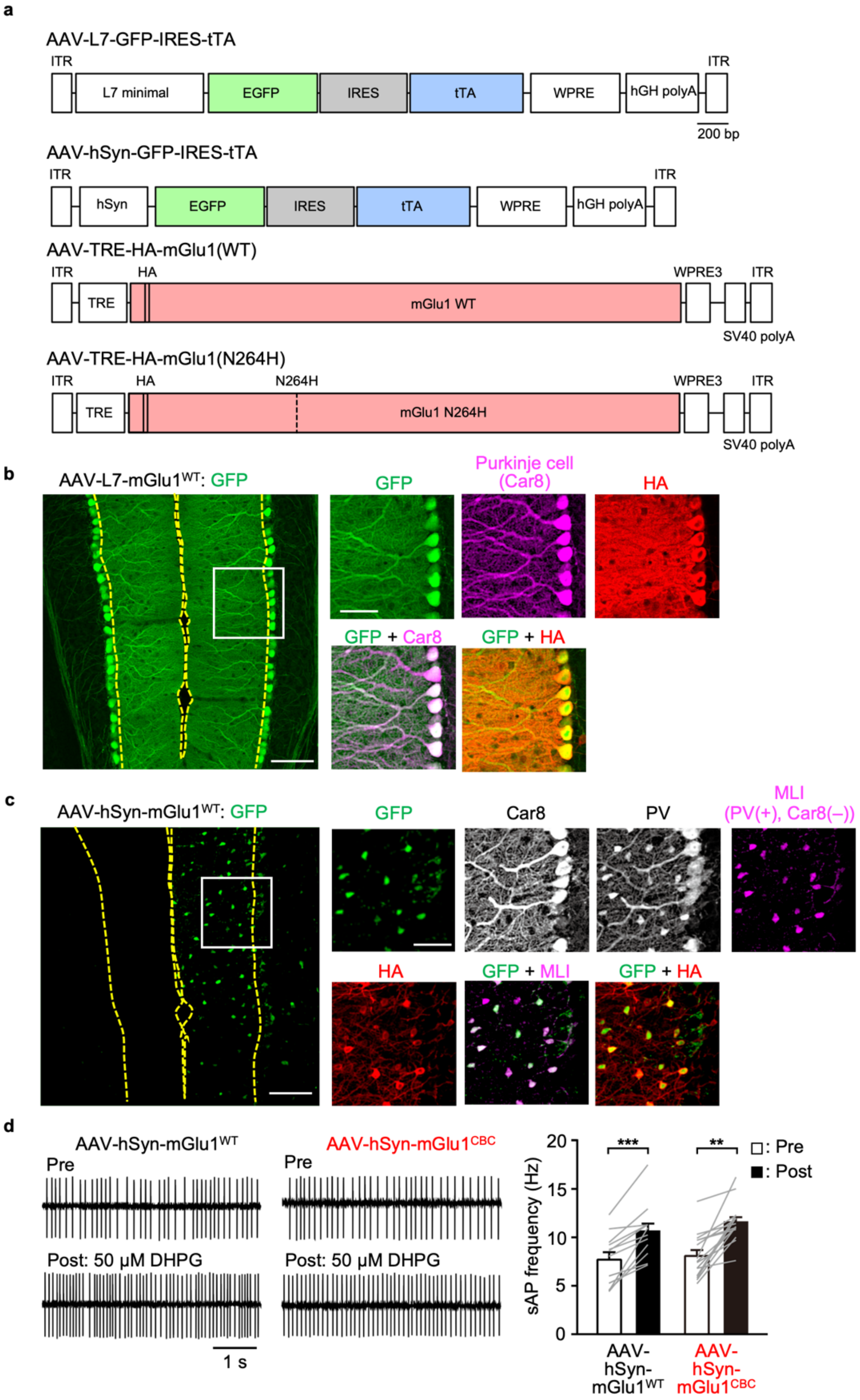


**Supplementary Figure 14 | AAV-mediated expression of mGlu1 in the cerebellum for dA-CBC.** (**a**) AAV vector constructs used in this study. (**b**) Confocal microscopic images of immune-positive signals for GFP, Car8 and HA-tag in the cerebellar slice infected with AAV-L7-mGlu1$^{WT}$. Scale bars, 100 μm (in a large left panel) and 50 μm (in small right panels). (**c**) Confocal microscopic images of immune-positive signals for GFP, Car8, PV and HA-tag in the cerebellar slice infected with AAV-hSyn-mGlu1$^{WT}$. The white squarer ROI is expanded on the right. To obtain the MLI image, immunofluorescent signals in the Car8 image were subtracted from that in the PV image. Scale bars, 100 μm (in a large left panel) and 50 μm (in small right panels). (**d**) sAP-mediated currents of AAV-hSyn-mGlu1$^{WT}$ and AAV-hSyn-mGlu1$^{CBC}$ -infected MLIs with bath application of 50 μM DHPG. Upper: sAP-mediated currents before DHPG treatment. Lower: sAP-mediated currents after DHPG treatment. [n = 15 cells from 4 mice (AAV-hSyn-mGlu1$^{WT}$, $P = 6.550 \times 10^{-4}$) or 12 cells

from 4 mice (AAV-hSyn-mGlu1$^{CBC}$, $P$ = 0.002)]. (two-sided Wilcoxon signed-rank test ***$P < 0.001$, **$P < 0.01$). Data are presented as mean ± s.e.m.

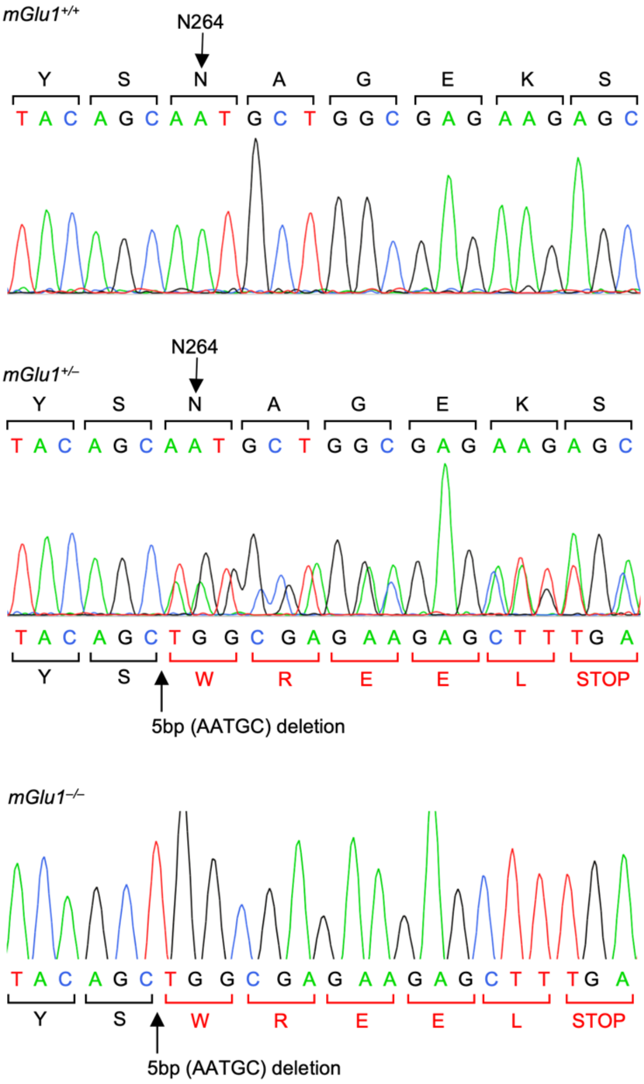


**Supplementary Figure 15 | Sequence analyses of exon 3 of mGlu1 using *mGlu1*$^{+/+}$, *mGlu1*$^{+/–}$, or *mGlu1*$^{–/–}$ genome.**

## Supplementary Methods

### Inositol-1-phosphate (IP1) assay

HEK293 cells were transfected with plasmids (mGlu1(N264H) mutant or control vector) using a Neon transfection system (Thermo Fisher Scientific) according to the manufacturer's instruction. The transfected cells were seeded into a 24-well plate at $1.0\times10^5$ cells and incubated at 37 °C and 5% $CO_2$ for 2 days, and then the culture medium was exchanged to HBS and incubated for 3 h. The cells were stimulated by 100 µM of glutamate or 10 µM of Pd(bpy) in HBS supplemented with 20 mM LiCl at 37 °C for 1 h. IP1 accumulation was measured using IP-One Gq Kit (Cisbio) according to the manufacturer's instruction. The assay was performed into white 96-well plate (Thermo Fisher Scientific). The Homogeneous Time-Resolved Fluorescence (HTRF) signal was read on a FlexStation 3 (Molecular Devices). Data acquisition and analysis were performed using SoftMax Pro software (Molecular Devices).

### Gross cerebellar anatomy

To observe gross structure in adult mouse cerebella, cresyl violet-staining was performed, as previously described[S1]. Bright-field images were captured using a CCD camera (DP70, Olympus) attached to a stereomicroscope (SMZ1000, Nikon).

### Rota-rod test

To evaluate motor coordination of *mGlu1*$^{+/+}$, *mGlu1*$^{CBC/CBC}$ or *mGlu1*$^{-/-}$ mice, accelerating rota-rod test was performed at 4−40 rpm using a single lane rota-rod treadmill (MK-630B, Muromachi Kikai Co., Ltd.), and the time that each mouse stayed on the rod was measured.

## Synthesis and Characterization

### General materials and methods for organic synthesis

All chemical reagents and solvents were purchased from commercial sources (FUJIFILM Wako pure chemical, TCI chemical, Sigma-Aldrich) and were used without further purification. Thin layer chromatography (TLC) was performed on silica gel 60 F254 precoated aluminum sheets (Merck). Chromatographic purification was performed using flash column chromatography on silica gel 60 N (neutral, 40–50 µm, Kanto Chemical). $^1$H-NMR or $^{13}$C-NMR spectra were recorded in deuterated solvents on an Advance III HD 500 MHz or Advance III HD 300 MHz (Bruker). Chemical shifts were referenced to residual solvent peaks or tetramethylsilane ($\delta$ = 0 ppm). Multiplicities are abbreviated as follows: s = singlet, d = doublet, t = triplet, m = multiplet, brs = broad singlet. High resolution mass spectra were measured on a compact (Bruker) equipped with electron spray ionization (ESI). Reversed-phase HPLC (RP-HPLC) was carried out on a Hitachi Chromaster system equipped with a UV detector, and an YMC-Pack ODS-A column.

## Synthesis of Compound FITM-Cy3

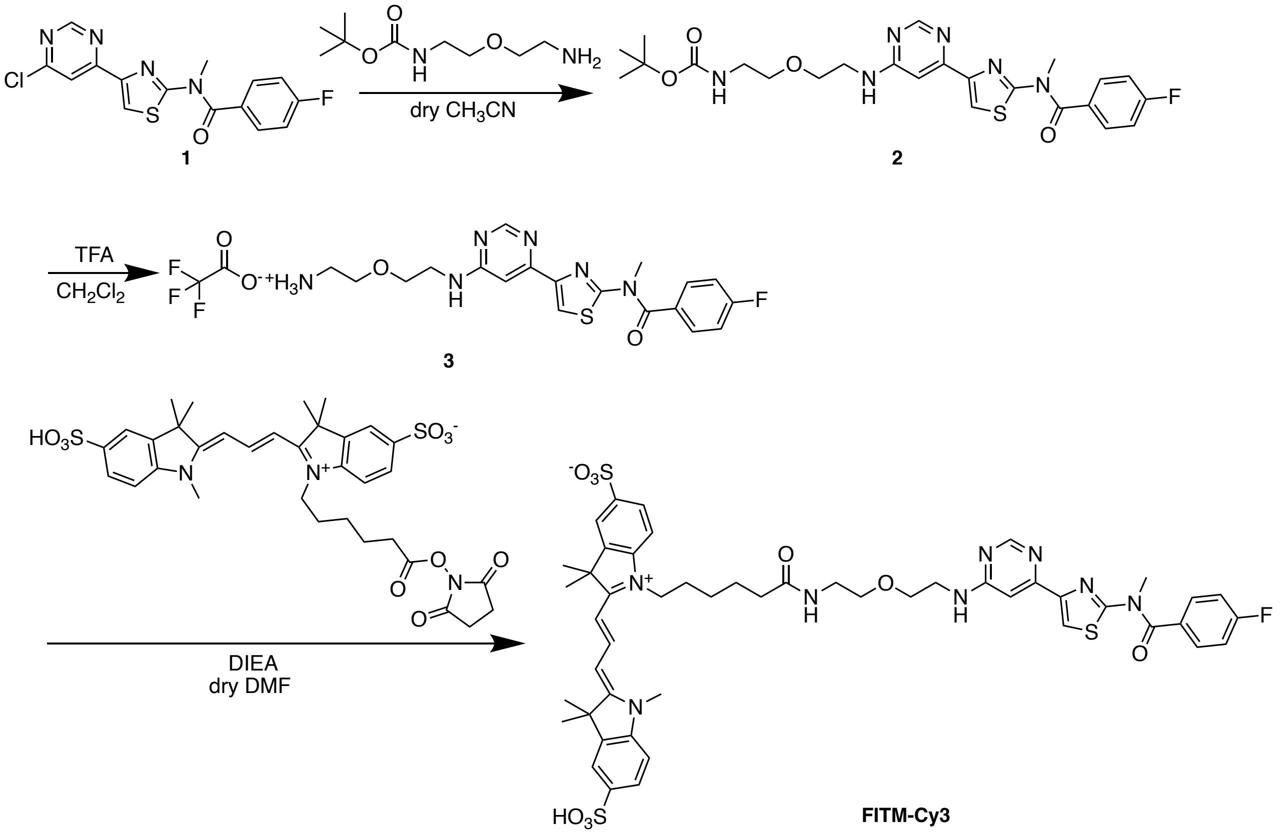

Synthesis of Compound **2**

*N*-(*tert*-Butoxycarbonyl)-2-(2-aminoethoxy) ethylamine (ref. S2) (50 mg, 0.25 mmol) was added to a solution of **1** (ref. S3) (30 mg, 86 μmol) in dry $CH_3CN$ (5 mL). The reaction mixture was stirred for 61 h in a $N_2$ atmosphere at 80 °C. The reaction mixture was evaporated to distil away $CH_3CN$, and $CHCl_3$ was added to the residue. The mixture was washed with saturated $NH_4Cl$ aqueous solution and brine. The organic layer was dried with $Na_2SO_4$ and concentrated *in vacuo*. The residue was purified by column chromatography (silica gel, EtOAc/n-hexane = 2/1) to afford compound **2** (33 mg, 0.064 mmol, 74% yield) as a colorless amorphous substance. $^1$H-NMR (500 MHz, $CDCl_3$) $\delta$ 8.59 (s, 1H), 7.93 (s, 1H), 7.61 (dd, $J$ = 5.3, 8.8 Hz, 2H), 7.20 (t, $J$ = 8.8 Hz, 2H), 7.13 (d, $J$ = 1.0 Hz, 1H), 5.54 (brs, 1H, NH), 4.97 (brs, 1H, NH), 3.74 (s, 3H), 3.68 (brt, $J$ = 4.2 Hz, 2H), 3.66-3.60 (m, 2H), 3.55 (brt, $J$ = 5.1 Hz, 2H), 3.36-3.30 (m, 2H), 1.44 (s, 9H); $^{13}$C-NMR (126 MHz, $CDCl_3$) $\delta$ 169.6, 165.3, 163.3, 160.5, 158.6, 156.2, 132.6, 131.0, 130.6, 130.3, 128.9, 116.1, 79.6, 70.4, 69.7, 68.3, 41.1, 40.5, 38.6, 28.5; HRMS (ESI$^+$): calcd for $[M + Na]^+$ ($C_{24}H_{29}FN_6NaO_4S$) 539.1847, found 539.1851.

Synthesis of **FITM-Cy3**

Trifluoroacetic acid (200 μL, 298 mg, 2.6 mmol) was added to a solution of **2** (28 mg, 54 μmol) in $CH_2Cl_2$ (1 mL). The reaction solution was stirred for 1 h at room temperature. After confirming the consumption of **2**, the reaction solution was concentrated *in vacuo* to obtain **3**. Sulfo-Cy3 NHS ester

(1 mg, 1.3 μmol) and *N*,*N*-diisopropylethylamine (5 μl, 3.7 mg, 28.7 μmol) were added to a solution of **3** (5.0 mg, 9.4 μmol) in dry DMF (500 μL). The reaction solution was stirred overnight in a $N_2$ atmosphere at room temperature. The residue was purified by RP-HPLC (ODS-A, 250 x 10 mm, mobile phase; $CH_3CN$: 10 mM $AcONH_4$ aq. = 5:95 (0 min), 65:35 (60 min), flow rate; 3.0 mL/min, detection; UV (220 nm)) to afford FITM-Cy3 (0.58 μmol determined using the molecular extinction coefficient of Sulfo-Cy3 NHS ester (162,000 $M^{-1}cm^{-1}$), 45% yield) as a magenta powder. $^{1}$H NMR (500 MHz, $CD_3OD$) $\delta$ 8.52 (t, *J* = 13.5 Hz, 1H), 8.38 (s, 1H), 7.95 (d, *J* = 1.6 Hz, 1H), 7.94 (d, *J* = 1.6 Hz, 1H), 7.91 (dd, *J* = 1.6, 8.3 Hz, 1H), 7.91 (dd, *J* = 1.6, 8.3 Hz, 1H), 7.89 (s, 1H), 7.68 (dd, *J* = 5.3, 8.8 Hz, 2H), 7.38 (d, *J* = 8.3 Hz, 1H), 7.34 (d, *J* = 8.3 Hz, 1H), 7.29 (t, *J* = 8.8 Hz, 2H), 7.28 (d, *J* = 0.9 Hz, 1H), 6.45 (d, *J* = 13.5 Hz, 1H), 6.40 (d, *J* = 13.4 Hz, 1H), 4.09 (brt, *J* = 7.5 Hz, 2H), 3.72 (s, 3H), 3.69 (s, 3H), 3.64 (brt, J = 4.5 Hz, 2H), 3.62-3.56 (m, 2H), 3.52 (t, *J* = 5.5 Hz, 2H), 3.34 (t, *J* = 5.5 Hz, 2H), 2.18 (brt, *J* = 6.4 Hz, 2H), 1.83-1.76 (m, 2H), 1.71-1.63 (m, 2H), 1.45-1.38 (m, 2H); HRMS ($ESI^-$): calcd for $[M-H]^-$ ($C_{49}H_{54}FN_8O_9S_3$) 1013.3165, found 1013.3163.

**<u>Synthesis of Pd(sulfo-bpy)</u>**

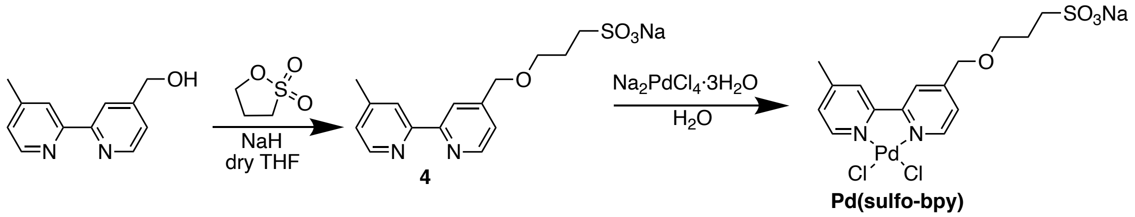


Synthesis of compound **4**

A solution of 4-hydroxymethyl-4'-methyl-2,2'-bipyridine (100 mg, 0.5 mmol) and sodium hydride (24 mg, 1.0 mmol) in dry THF (5 mL) was stirred on ice for 30 min under $N_2$ atmosphere. 1,3-propane sultone 73 mg (0.6 mmol) was added to the solution and stirred for 14 h at room temperature. After removal of the solvent by evaporation, the crude was purified by RP-HPLC (ODS-A, 250 x 10 mm, mobile phase; $CH_3CN$: 10 mM $AcONH_4$ aq. = 5:95 (0 min), 5:95 (10 min), 40:60 (60 min), flow rate; 3.0 mL/min, detection; UV (220 nm)) and neutrized with 1M NaOH, giving compound **4** (60 mg, 0.17 mmol, 34%) as a colorless oil. $^{1}$H-NMR (500 MHz, $CD_3OD$) $\delta$ 8.61 (d, *J* = 5.0 Hz, 1H), 8.51 (d, *J* = 5.0 Hz, 1H), 8.23 (s, 1H), 8.15 (s, 1H), 7.46 (d, *J* = 5.0 Hz, 1H), 7.34(d, *J* = 5.0 Hz, 1H), 4.66 (s, 2H), 3.71 (t, *J* = 6.3 Hz, 2H), 3.00-2.96 (m, 2H), 2.48 (s, 3H), 2.18-2.13 (m, 2H). $^{13}$C-NMR (126 MHz, $D_2O$) $\delta$ 155.8, 150.9, 150.4, 150.0, 148.7, 144.8, 126.6, 123.9, 120.6, 120.5, 70.3, 69.2, 47.8, 24.4, 21.0.

Synthesis of **Pd(sulfo-bpy)**

A solution of compound **4** (55 mg, 160 μmol) in $H_2O$ (3.0 mL) was added to a solution of sodium tetracholoropalladate trihydrate (II) (56 mg, 160 μmol) in $H_2O$ (2.0 mL). The solution was stirred at

room temperature for 12 h. After concentration of the solution to 0.5 ml by evaporation, crystals were obtained from vapor diffusion of acetone into a $H_2O$ at room temperature, giving Pd(sulfo-bpy) (49 mg, 94 μmol, 59%) as an orange solid. $^1$H-NMR (500 MHz, $CD_3OD$) $\delta$ 9.10 (s, 1H), 9.00 (s, 1H), 8.39 (s, 1H), 8.36 (s, 1H), 7.63 (d, $J$ = 6.0 Hz, 1H), 7.52 (d, $J$ = 6.0 Hz, 1H), 4.76 (s, 2H), 3.78 (t, $J$ = 6.0 Hz, 2H), 2.99 (t, $J$ = 7.5 Hz, 2H), 2.20-2.14 (m, 2H). $^{13}$C-NMR (126 MHz, $D_2O$): $\delta$ 155.1, 154.9, 154.4, 154.2, 149.2, 148.5, 128.1, 125.2, 124.6, 121.6, 69.8, 69.6, 48.0, 24.5, 21.1.

**Synthesis of Pd(EG-bpy)**

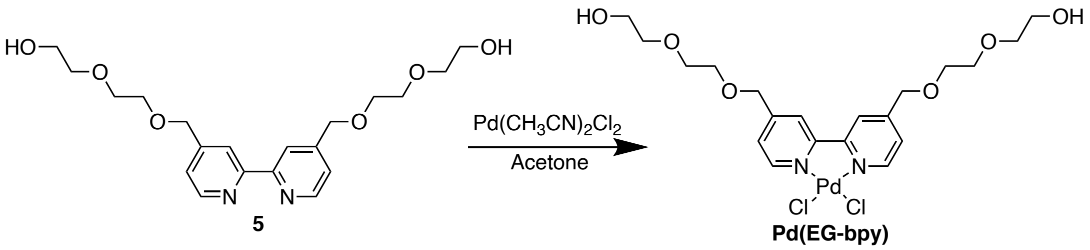


A solution of compound **5** (ref. S4) (10 mg, 26 μmol) in acetone (0.4 mL) was added to a solution of Bis(acetonitrile) dichloro palladiium (II) (6 mg, 23 μmol) in acetone. The solutioin was stood still at room temperature for 3 days, and filtered to obtain Pd(EG-bpy) (5.7 mg, 26 μmol, 59%) as an orange needle crystal. $^1$H-NMR (500 MHz, $CD_3OD$) $\delta$ 8.64 (d, $J$ = 5.0 Hz, 2H), 8.40 (s, 2H), 7.32 (m, 2H), 4.68 (s, 2H), 3.74 (t, J = 6.5 Hz, 4H), 2.98 (m, 4H), 2.17 (m, 4H); $^{13}$C-NMR (126 MHz, $CDCl_3$) $\delta$ 155.8, 153.8, 149.9, 124.1, 121.6, 72.7, 70.7, 70.5, 70.2, 61.6.

**Synthesis of Pd(di-sulfo-bpy)**

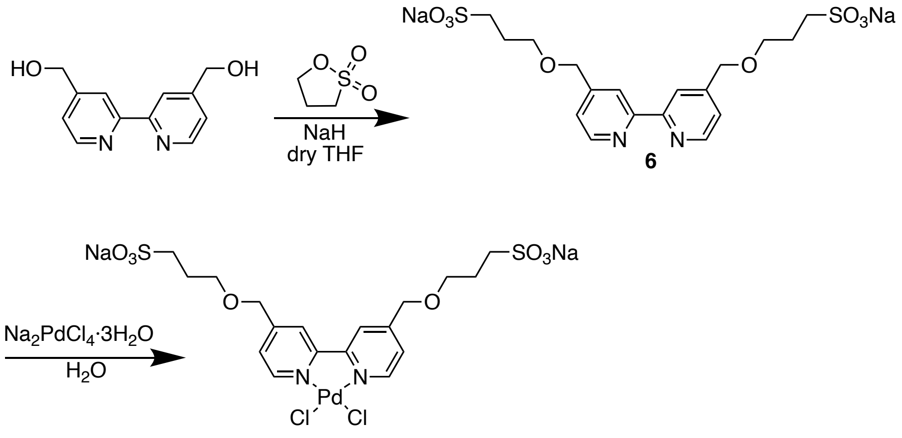

Pd(di-sulfo-bpy)

Synthesis of coumpound **6**

A solution of 4,4'-hydroxymethyl-2,2'-bipyridine (200 mg, 0.92 mmol) and sodium hydride (48 mg, 2.0 mmol) in dry THF (5 mL) was stirred on ice for 15 min under $N_2$ atmosphere. 1,3-propane sultone 244 mg (2.0 mmol) was added to the solution and stirred for 3 days at room temperature. The precipitation was collected and purified by RP-HPLC (ODS-A, 250 x 10 mm, mobile phase; $CH_3CN$: 10 mM $AcONH_4$ aq. = 0:100 (0 min), 0:100 (10 min), 40:60 (60 min), flow rate; 3.0 mL/min, detection; UV (220 nm)), and neutrized with 1M NaOH, giving compound **6** (60 mg, 0.17 mmol, 34%) as a colorless oil. $^1$H-NMR (500 MHz, $CD_3OD$) $\delta$ 8.63 (d, $J$ = 5.0 Hz, 2H), 8.29 (s, 2H), 7.48 (d, $J$ = 5.0 Hz, 2H), 4.69 (s, 2H), 3.74 (t, $J$ = 6.5 Hz, 4H), 2.98-2.96 (m, 4H), 2.20-2.14 (m, 4H); $^{13}$C-NMR (126 MHz, $D_2O$) $\delta$ 154.9, 149.3, 149.0, 122.8, 120.4, 70.6, 69.1, 47.9, 24.4.

Synthesis of **Pd(di-sulfo-bpy)**

A solution of compound **6** (45 mg, 89 μmol) in MeOH (2.2 mL) was added to a solution of sodium tetracholoropalladate trihydrate (II) (23 mg, 89 μmol) in MeOH (12 mL). The solution was stirred at room temperature for 1 h. After removal of the solvent by evaporation, the solid was dissolved in MeOH, and reprecipitated with diethylether, giving Pd(di-sulfo-bpy) (18 mg, 26 μmol, 30%) as an orange solid. $^1$H-NMR (300 MHz, $CD_3OD$) $\delta$ 9.14 (d, $J$ = 6.0 Hz, 2H), 8.40 (s, 2H), 7.70 (d, $J$ = 6.6 Hz, 2H), 4.77 (s, 2H), 3.77 (t, $J$ = 6.6 Hz, 4H), 3.00-2.95 (m, 4H), 2.20-2.11 (m, 4H); $^{13}$C-NMR (126 MHz, $D_2O$): $\delta$ 155.6, 154.2, 149.5, 124.8, 121.9, 69.9, 69.6, 48.0, 24.5.

**Supplementary references**

S1. Kakegawa, W. *et al.* Anterograde C1ql1 signaling is required in order to determine and maintain a single-winner climbing fiber in the mouse cerebellum. *Neuron* **85**, 316–329 (2015).

S2. Traquete, R. *et al.* Evaluation of linker length effects on a BET bromodomain probe. *Chem. Commun.* **55**, 10128-10131 (2019).

S3. Satoh, A. *et al.* Discovery and in vitro and in vivo profiles of 4-fluoro-*N*-[4-[6-(isopropylamino)pyrimidin-4-yl]-1,3-thiazol-2-yl]-*N*-methylbenzamide as novel class of an orally active metabotropic glutamate receptor 1 (mGluR1) antagonist. *Bioorg. Med. Chem. Lett.* **19**, 5464-5468 (2009).

S4. Miyoshi, D. *et al.* Artificial G-Wire Switch with 2,2'-Bipyridine Units Responsive to Divalent Metal Ions. *J. Am. Chem. Soc.* **129**, 5919–5925 (2007).
