# Supplementary material for: Coordination chemogenetics for activation of GPCR-type glutamate receptors in brain tissue
Source: Nat Commun. 2022 Jun 16;13:3167. doi: 10.1038/s41467-022-30828-0 (PMC9203742; doi:10.1038/s41467-022-30828-0)
Supplement: Supplementary file 3 — Description of Additional Supplementary Files [file 41467_2022_30828_MOESM3_ESM.pdf]

## Description of Additional Supplementary Files

**Supplementary Movie 1:** *mGlu1*$^{CBC/CBC}$ mice do not show the ataxic phenotype observed in *mGlu1* $^{-/-}$ mice.
