# Supplementary material for: Coordination chemogenetics for activation of GPCR-type glutamate receptors in brain tissue
Source: Nat Commun. 2022 Jun 16;13:3167. doi: 10.1038/s41467-022-30828-0 (PMC9203742; doi:10.1038/s41467-022-30828-0)
Supplement: Supplementary file 5 — Reporting Summary [file 41467_2022_30828_MOESM5_ESM.pdf]

nature portfolio

Corresponding author(s): Michisuke Yuzaki, Itaru Hamachi, Shigeki Kiyonaka

Last updated by author(s): 2022-4-24

Reporting Summary

Nature Portfolio wishes to improve the reproducibility of the work that we publish. This form provides structure for consistency and transparency in reporting. For further information on Nature Portfolio policies, see our Editorial Policies and the Editorial Policy Checklist.

Statistics

For all statistical analyses, confirm that the following items are present in the figure legend, table legend, main text, or Methods section.

n/a	Confirmed	
☐	☒	The exact sample size (*n*) for each experimental group/condition, given as a discrete number and unit of measurement
☐	☒	A statement on whether measurements were taken from distinct samples or whether the same sample was measured repeatedly
☐	☒	The statistical test(s) used AND whether they are one- or two-sided<br>*Only common tests should be described solely by name; describe more complex techniques in the Methods section.*
☒	☐	A description of all covariates tested
☒	☐	A description of any assumptions or corrections, such as tests of normality and adjustment for multiple comparisons
☐	☒	A full description of the statistical parameters including central tendency (e.g. means) or other basic estimates (e.g. regression coefficient) AND variation (e.g. standard deviation) or associated estimates of uncertainty (e.g. confidence intervals)
☐	☒	For null hypothesis testing, the test statistic (e.g. *F*, *t*, *r*) with confidence intervals, effect sizes, degrees of freedom and *P* value noted<br>*Give P values as exact values whenever suitable.*
☒	☐	For Bayesian analysis, information on the choice of priors and Markov chain Monte Carlo settings
☒	☐	For hierarchical and complex designs, identification of the appropriate level for tests and full reporting of outcomes
☒	☐	Estimates of effect sizes (e.g. Cohen's *d*, Pearson's *r*), indicating how they were calculated

Our web collection on statistics for biologists contains articles on many of the points above.

Software and code

Policy information about availability of computer code

Data collection	AQUACOSMOS 2.6 (HAMAMATSU PHOTONICS), Evolution Capt 18.04 (Vilber Lourmat), ZEN 3.1 blue software (Zeiss), UVProbe 2.70 (SHIMADZU), SoftMax Pro7.1.2 (Molecular Devices), pClamp 9.2 (Molecular Devices)
Data analysis	KaleidaGraph 4.5J (Synergy software), Image J software version 1.52k, Microsoft Excel2019

For manuscripts utilizing custom algorithms or software that are central to the research but not yet described in published literature, software must be made available to editors and reviewers. We strongly encourage code deposition in a community repository (e.g. GitHub). See the Nature Portfolio guidelines for submitting code & software for further information.

Data

Policy information about availability of data

All manuscripts must include a data availability statement. This statement should provide the following information, where applicable:

- Accession codes, unique identifiers, or web links for publicly available datasets
- A description of any restrictions on data availability
- For clinical datasets or third party data, please ensure that the statement adheres to our policy

The authors declare that the all source data are provided as a Source Data file. All data supporting the findings of this study are available in the article and its Supplementary information file.

Field-specific reporting

Please select the one below that is the best fit for your research. If you are not sure, read the appropriate sections before making your selection.

- [x] Life sciences
- [ ] Behavioural & social sciences
- [ ] Ecological, evolutionary & environmental sciences

For a reference copy of the document with all sections, see nature.com/documents/nr-reporting-summary-flat.pdf

Life sciences study design

All studies must disclose on these points even when the disclosure is negative.

Sample size	Sample size are indicated in the figure legends. No statistical methods were used to predetermine sample sizes. The sample size was determined based on the previous studies in the same research fields.
Data exclusions	No data were excluded from the analyses.
Replication	All experiments were replicated at least three times with similar observations.
Randomization	Randomization was not relevant to this study.
Blinding	The work did not involve a study that required blinding because no group allocation was involved in this study.

Reporting for specific materials, systems and methods

We require information from authors about some types of materials, experimental systems and methods used in many studies. Here, indicate whether each material, system or method listed is relevant to your study. If you are not sure if a list item applies to your research, read the appropriate section before selecting a response.

Materials & experimental systems

n/a	Involved in the study	
[ ]	[x]	Antibodies
[ ]	[x]	Eukaryotic cell lines
[x]	[ ]	Palaeontology and archaeology
[ ]	[x]	Animals and other organisms
[x]	[ ]	Human research participants
[x]	[ ]	Clinical data
[x]	[ ]	Dual use research of concern

Methods

n/a	Involved in the study	
[x]	[ ]	ChIP-seq
[x]	[ ]	Flow cytometry
[x]	[ ]	MRI-based neuroimaging

Antibodies

Antibodies used

mGluR1 (BD biosciences, catalog number: 610964),
mGluR1a (Frontier Institute, catalog number: MSFR104040),
GluD2 (Frontier Institute, catalog number: MSFR102610),
pan AMPAR (Frontier Institute, catalog number: MSFR104670),
beta actin (MBL, catalog number: M177-3),
calbindin (Swant, catalog number: 300),
bassoon (Enzo life sciences, catalog number: ADI-VAM-PS003),
Car8 (Frontier Institute, catalog number: MSFR100510)
PV (Frontier Institute, catalog number: MSFR105230)
GFP (Millipore, catalog number AB16901)
HA (CST, catalog number 3724)
rabbit IgG HRP (Cytiva, catalog number: NA934-1ML),
mouse IgG HRP (MBL, catalog number: 300),
rabbit IgG Alexa Fluor 488 (Thermofisher, catalog number: A21206),
rabbit IgG Cy3 (Jackson ImmunoResearch, catalog number: 711-165-152)
guinea pig IgG Alexa Fluor 488 (Jackson ImmunoResearch, catalog number: 706-545-148)
guinea pig IgG DyLight405 (Jackson ImmunoResearch, catalog number: 706-475-148)
guinea pig IgG HRP (Millipore, catalog number AP108P)
chicken IgY Alexa Fluor 488 (JacksonImmunoResearch, catalog number: 703-545-155)
goat IgG Alexa Fluor Plus 647 (Thermofisher, catalog number: A32849)

Validation

All of antibodies were commercially available and commonly used. All antibody were verified by the supplier and each lot has been

quality tested. Validation details of the antibodies are available on the manufacture's websites as follows.
mGluR1; https://www.bdbiosciences.com/content/dam/bdb/products/global/reagents/microscopy-imaging-reagents/immunofluorescence-reagents/610965_base/pdf/610964.pdf
mGluR1a; https://nittobo-nmd.co.jp/pdf/reagents/mGluR1a.pdf
GluD2; https://nittobo-nmd.co.jp/pdf/reagents/GluD2-C.pdf
pan AMPAR; https://nittobo-nmd.co.jp/pdf/reagents/panAMPAR.pdf
beta actin; https://ruo.mbl.co.jp/bio/dtl/dtlfiles/M177-3-ver4.pdf
calbindin; https://www.swant.com/pdfs/Monoclonal_anti_calbindin_d28k_300.pdf
bassoon; https://www.enzolifesciences.com/ADI-VAM-PS003/bassoon-monoclonal-antibody-sap7f407/
Car8; https://nittobo-nmd.co.jp/pdf/reagents/Car8.pdf
PV; https://nittobo-nmd.co.jp/pdf/reagents/PV.pdf
GFP; https://www.merckmillipore.com/JP/ja/product/Anti-Green-Fluorescent-Protein-Antibody,MM_NF-AB16901
HA; https://www.cellsignal.jp/products/primary-antibodies/ha-tag-c29f4-rabbit-mab/3724
rabbit IgG Alexa Fluor 488; https://www.thermofisher.com/antibody/product/Donkey-anti-Rabbit-IgG-H-L-Highly-Cross-Adsorbed-Secondary-Antibody-Polyclonal/A-21206
rabbit IgG Cy3; https://www.jacksonimmuno.com/catalog/products/711-165-152
guinea pig IgG Alexa Fluor 488; https://www.jacksonimmuno.com/catalog/products/706-545-148
guinea pig IgG DyLight405; https://www.jacksonimmuno.com/catalog/products/706-475-148
chicken IgY Alexa Fluor 488; https://www.jacksonimmuno.com/catalog/products/703-545-155
goat IgG Alexa Fluor Plus 647; https://www.thermofisher.com/antibody/product/Donkey-anti-Goat-IgG-H-L-Highly-Cross-Adsorbed-Secondary-Antibody-Polyclonal/A32849

Eukaryotic cell lines

Policy information about cell lines

Cell line source(s)	HEK293 cell and PC12 cell lines were purchased from ATCC (CRL-1573 and CRL-1721). 293AAV cell line was purchased from Cell Biolabs (AAV-100).
Authentication	Cell line was authenticated by the ATCC or Cell Biolabs, cell lines were not authenticated within the lab.
Mycoplasma contamination	Mycoplasma detection was teated negative.
Commonly misidentified lines (See ICLAC register)	No commonly misidentified cell lines were used in this study.

Animals and other organisms

Policy information about studies involving animals; ARRIVE guidelines recommended for reporting animal research

Laboratory animals	We used ICR mice (aged 3–5 weeks or pregnant) and C57BL/6J wild-type mice (aged 3–5 weeks) provided by Japan SLC and mGlu1 knock-in mice of both sexes. All animals were housed in a controlled environment (12 h light/dark cycle at 25 °C with 40–60% humidity) and had free access to food and water,
Wild animals	The study did not involve wild animals.
Field-collected samples	The study did not involve samples collected form the field.
Ethics oversight	All experiment procedures were performed in accordance with the National Institute of Health Guide for the Care and Use of Laboratory Animals and approved by the Institution Animal Use Committees of Nagoya University, Kyoto University, Keio University and Tsukuba University.

Note that full information on the approval of the study protocol must also be provided in the manuscript.
